# Body size, modifying factors, and postmenopausal breast cancer risk in a multiethnic population: the San Francisco Bay Area Breast Cancer Study

**DOI:** 10.1186/2193-1801-2-239

**Published:** 2013-05-24

**Authors:** Esther M John, Amanda I Phipps, Meera Sangaramoorthy

**Affiliations:** Cancer Prevention Institute of California, 2201 Walnut Ave, Suite 300, Fremont, CA 94538 USA; Division of Epidemiology, Department of Health Research and Policy, and Stanford Cancer Institute, Stanford University School of Medicine, Stanford, CA 94305 USA; Division of Public Health Sciences, Fred Hutchinson Cancer Research Center, Seattle, WA 98109 USA

**Keywords:** African Americans, Body size, Breast cancer, Estrogen receptor, Hispanics, Progesterone receptor

## Abstract

Data on body size and postmenopausal breast cancer in Hispanic and African American women are inconsistent, possibly due to the influence of modifying factors. We examined associations between adiposity and risk of breast cancer defined by hormone receptor status in a population-based case-control study conducted from 1995–2004 in the San Francisco Bay Area. Multivariate adjusted odds ratios and 95% confidence intervals were calculated using unconditional logistic regression. Associations with body size were limited to women not currently using menopausal hormone therapy (801 cases, 1336 controls). High young-adult body mass index (BMI) was inversely associated with postmenopausal breast cancer risk, regardless of hormone receptor status, whereas high current BMI and high adult weight gain were associated with two-fold increased risk of estrogen receptor and progesterone receptor positive breast cancer, but only in women with a low young-adult BMI (≤22.4 kg/m^2^) or those with ≥15 years since menopause. Odds ratios were stronger among non-Hispanic Whites than Hispanics and African Americans. Waist circumference and waist-to-height ratio increased breast cancer risk in Hispanics and African Americans only, independent of BMI. These findings emphasize the importance of considering tumor hormone receptor status and other modifying factors in studies of racially/ethnically diverse populations.

## Introduction

Obesity has long been recognized as a risk factor for postmenopausal breast cancer (BC) in studies of primarily non-Hispanic White (NHW) women (World Cancer Research Fund / American Institute for Cancer Research 2007). Only three studies in Hispanics (Wenten et al. 2002; Slattery et al. 2007; White et al. 2012) and eight studies in African Americans (AA) (White et al. 2012; Austin et al. 1979; Schatzkin et al. 1987; Adams-Campbell et al. 1996; Hall et al. 2000; Zhu et al. 2005; Palmer et al. 2007; Berstad et al. 2010) examined the relation between obesity and postmenopausal BC risk, and some of their findings contradict those reported for NHW women, suggesting differences in effects by racial/ethnic groups (Slattery et al. 2007; Sexton et al. 2011). In NHWs, BC risk is increased by 3-5% both per 2 kg/m^2^ increase in body mass index (BMI) and per 5 kg of weight gain (World Cancer Research Fund / American Institute for Cancer Research 2007). Young-adult obesity, on the other hand, has been associated with reduced postmenopausal BC risk, both in cohort (Barnes-Josiah et al. 1995; Huang et al. 1997; Morimoto et al. 2002; Ahn et al. 2007) and case–control (Berstad et al. 2010; Chu et al. 1991; Brinton & Swanson 1992; Magnusson et al. 1998) studies. A number of factors appear to modify the relation with body size. Increased risks associated with BMI and weight gain may be limited to women with a low young-adult BMI (Chu et al. 1991; Canchola et al. 2012). Stronger associations with body size have been found in women with estrogen receptor and progesterone receptor positive (ER+PR+) BC (Potter et al. 1995; Huang et al. 2000; Colditz et al. 2004; Suzuki et al. 2009), those not using menopausal hormone therapy (HT) (Morimoto et al. 2002; Friedenreich 2001; Feigelson et al. 2004), or with longer time since menopause (Chu et al. 1991; Magnusson et al. 1998; Macinnis et al. 2004). Data on abdominal obesity and BC risk in postmenopausal women are also inconsistent (World Cancer Research Fund / American Institute for Cancer Research 2007) and uncertainties remain whether associations are independent of overall obesity or differ by race/ethnicity.

We report on the relation between overall and abdominal adiposity and risk of postmenopausal BC defined by hormone receptor status in a multiethnic population, and the role of modifying factors.

## Materials and methods

### Study population

The San Francisco Bay Area Breast Cancer Study, a population-based case-control study (John et al. 2003; John et al. 2005), identified 17,581 women aged 35–79 years with newly diagnosed invasive BC through the Greater Bay Area Cancer Registry. Following telephone screening on study eligibility (83% participation), 2,571 cases were selected (all Hispanics diagnosed from 1995–2002, all AAs diagnosed from 1995–1999, and a 10% random sample of NHWs diagnosed from 1995–1999). An in-person interview was completed by 2,258 (88%) cases, including 1,119 (89%) Hispanics, 543 (87%) AAs, and 596 (86%) NHWs.

Population controls, identified through random-digit dialing, were frequency-matched on race/ethnicity and 5-year age group (John et al. 2003). Of 3,170 eligible controls, 2,706 (85%) completed the in-person interview, including 1,462 (88%) Hispanics, 598 (82%) AAs, and 646 (83%) NHWs.

This analysis was restricted to postmenopausal women (1,389 cases, 1,644 controls). Women were considered postmenopausal if their periods had stopped more than one year prior to diagnosis (cases) or selection into the study (controls), if they reported a bilateral oophorectomy, or if they were aged ≥55 years at the time of diagnosis/selection and had either started hormone therapy prior to the cessation of menses or had had a simple hysterectomy (without oophorectomy).

### Data collection

Information on adult height, weight in the reference year (defined as the calendar year before diagnosis for cases or before selection into the study for controls), young-adult weight, and other BC risk factors was collected using a structured questionnaire, administered in English or Spanish. Young-adult weight was based on reported weight at age 25–30 years for cases diagnosed before May 1998 and their matched controls, and on reported weight at age 20–29 years for cases diagnosed in May 1998 or later and their matched controls. Interviewers also took measurements of weight, height, waist and hip circumference (described in (John et al. 2010)). Lifetime physical activity was assessed, as described elsewhere (John et al. 2003). Usual dietary intake and alcohol consumption during the reference year was assessed by a modified version of the Block food frequency questionnaire (Block et al. 1986). Cancer registry information on ER and PR status was available for 85% of cases. The study was approved by the Institutional Review Board of the Cancer Prevention Institute of California and participants provided written informed consent.

### Body size variables

Current BMI was calculated as weight (kg) divided by height squared (m^2^), based on measured height at interview (or self-reported height for 10% of cases and 9% of controls who declined height measurements) and self-reported weight in the reference year (or measured weight at interview for 1% of cases and 3% of controls without self-report). BMI was classified as normal weight (<25.0 kg/m^2^), overweight (25.0-29.9 kg/m^2^) and obese (≥30.0 kg/m^2^) (WHO 2000). Underweight (BMI <18.5 kg/m^2^) women (9 cases, 16 controls) were grouped with normal weight women. Young-adult BMI was based on measured height at interview and self-reported weight in a woman’s twenties. Adult weight gain was calculated as the difference between self-reported young-adult weight and weight in the reference year. Waist-to-hip ratio (WHR) was calculated as a measure of body fat distribution reflecting both adipose tissue and muscle mass; waist-to-height ratio (WHtR) was calculated as a measure of visceral adiposity independent of height, which may more directly reflect abdominal adiposity (Molarius & Seidell 1998). WHR, WHtR, and waist and hip circumferences were categorized according to the tertile distribution among controls.

### Statistical analysis

Unconditional logistic regression was used to calculate odds ratios (OR) and 95% confidence intervals (CI) comparing cases to controls, both overall and separately for each racial/ethnic group. Polytomous logistic regression was used to compare ER+PR+ and ER-PR- case groups with a common control group. For all BCs combined and ER+PR+ BCs, multivariate analyses were adjusted for age (continuous) and factors significantly associated with BC risk in our study: birth place, education, first-degree family history of BC, personal history of benign breast disease, age at menarche, number of full-term pregnancies, age at first full-term pregnancy, lifetime breast-feeding, average lifetime physical activity, alcohol consumption, and caloric intake, categorized as shown in the tables. For ER-PR- BCs, analyses were adjusted for age, birth place, age at menarche, and lifetime breast-feeding. Analyses of all BCs combined were also adjusted for race/ethnicity. Analyses of current BMI, young-adult BMI and weight gain were mutually adjusted for each other. Linear trends were assessed across ordinal values of categorical variables. Significant differences in ORs between groups were tested using the Wald statistic *P* value. Two-sided *P* values are reported for tests of trend and interaction, with *P* values <0.05 considered statistically significant.

We assessed associations with current BMI and weight change within strata defined by median young-adult BMI (≤22.4 kg/m^2^, >22.4 kg/m^2^), median time since menopause (<15, ≥15 years), and, in analyses of abdominal adiposity, by current BMI (<25.0 kg/m^2^, ≥25.0 kg/m^2^). Primary analyses were restricted to women not currently using menopausal HT, as previous studies have found no associations with body size among current HT users (Huang et al. 1997; Morimoto et al. 2002). Current HT use was defined as starting HT prior to the year of diagnosis/selection and reported use during the year of diagnosis/selection. All other women were classified as non-current HT users. Time since menopause was calculated as the difference between age at menopause and age at diagnosis/selection. Age at menopause was based on self-report for women with natural menopause and age at bilateral oophorectomy for women with surgical menopause.

The final analysis was based on 2,884 postmenopausal women (1,316 cases, 1,568 controls) after excluding 34 cases and 50 controls with missing information on confounding variables and 39 cases and 26 controls with unreliable caloric intake (<600 kcal/day or >5,000 kcal/day). Statistical analyses were conducted using SAS version 9.3 software (SAS Institute, Inc., Cary, North Carolina).

## Results

Cases were more likely than controls to be U.S.-born, have a first-degree family history of BC, a personal history of benign breast disease, higher education, earlier menarche, fewer full-term pregnancies, a shorter duration of breast-feeding, lower lifetime physical activity, and higher alcohol consumption (Table 1).

**Table 1:** **Characteristics of postmenopausal cases and controls**

	Cases (n = 1,316)		Controls (n = 1,568)		***P*** value
	n	%^a^	n	%^a^	
Age (years)					
35–44	20	2	25	2	
45–54	199	15	260	17	
55–64	531	40	625	40	
65–74	407	31	509	33	
≥75	159	12	149	10	
Race/ethnicity					
Hispanic	614	47	804	51	
Non-Hispanic White	389	30	399	26	
African American	313	24	365	23	
Joint ER/PR status					
ER+PR+	714	54			
ER+PR-	168	13			
ER-PR+	21	2			
ER-PR-	204	16			
Missing	209	16			
Menopausal hormone therapy use					<0.01
Never	517	39	644	41	
Former	284	22	692	44	
Current	498	38	214	14	
Missing	17	1	18	1	
Place of birth					<0.01
U.S.-born^b^	1,014	77	1,036	66	
Foreign-born	302	23	532	34	
Education (years)					<0.01
Some high school or less	398	30	600	38	
High school or vocational/technical school graduate	373	28	426	27	
Some college	297	23	297	19	
College graduate	248	19	245	16	
Family history of breast cancer in first-degree relatives					<0.01
No	1,089	83	1,371	87	
Yes	227	17	197	13	
Personal history of biopsy-confirmed benign breast disease					0.01
No	1,016	77	1,274	81	
Yes	300	23	294	19	
Age at menarche					<0.01
≤11	304	23	327	21	
12	349	27	356	23	
13	319	24	387	25	
≥14	344	26	498	32	
Parity					<0.01
Nulliparous	169	13	145	9	
Parous	1,147	87	1,423	91	
Number of full-term pregnancies, parous women					<0.01
1	162	14	177	12	
2	309	27	303	21	
3	263	23	349	25	
≥4	413	36	594	42	
Age at first full-term pregnancy (years), parous women					<0.01
≤19	302	26	415	29	
20–24	462	40	564	40	
25–29	235	21	298	21	
≥30	148	13	146	10	
Lifetime breast-feeding (months), parous women					<0.01
0	541	47	547	38	
≤ 6	250	22	294	21	
7–12	97	8	148	10	
13–24	126	11	184	13	
≥25	133	12	250	18	
Lifetime physical activity^c^ (hours/week)					0.04
≤ 6.9	376	29	391	25	
7.0-14.1	342	26	390	25	
14.2-25.4	291	22	403	26	
≥25.5	307	23	384	25	
Alcohol consumption^d,e^ (g/day)					<0.01
0	763	58	975	62	
0.1-4.9	262	20	312	20	
5.0-9.9	68	5	91	6	
10.0-19.9	115	9	107	7	
≥20	108	8	83	5	
Total caloric intake^c,d,e^ (kcal/day)					0.05
≤ 1362	287	22	394	25	
1363–1798	343	26	385	25	
1799–2435	379	29	398	25	
≥2436	307	23	391	25	

Abbreviations: ER-, estrogen receptor–negative; ER+, estrogen receptor–positive; PR-, progesterone receptor–negative; PR+, progesterone receptor–positive.

^a^ Percentages may not add up to 100% due to rounding.

^b^ U.S.-born includes 43 cases and 34 controls born in westernized countries such as Canada, Europe, Australia, or New Zealand.

^c^ Quartiles among all postmenopausal controls.

^d^ In reference year.

^e^ Excludes 39 cases and 26 controls whose total caloric intake was <600 kcal/day or >5,000 kcal/day.

Body size characteristics differed by race/ethnicity (Table 2). Among controls, the proportion of currently obese women (BMI ≥30.0 kg/m^2^) was higher in AAs and Hispanics than in NHWs. High young-adult BMI was twice as common in Hispanics as in NHWs, whereas the proportion of women with high weight gain was similar in the two groups. The prevalence of high weight gain, large waist and hip circumferences, and high WHR was lowest in NHWs, intermediate in Hispanics, and more than twice as high in AAs than in NHWs.

**Table 2:** **Body size among control women by race/ethnicity**

	Hispanics (n = 804)		African Americans (n = 365)		Non-Hispanic Whites (n = 399)		***P*** value^a^
	n	%^b^	n	%^b^	n	%^b^	
Current BMI (kg/m^2^) ^c^							* †
<25.0	147	18	80	22	185	47	
25.0-29.9	310	39	124	34	116	29	
≥30.0	342	43	159	44	97	24	
Young-adult BMI (kg/m^2^) ^d,e^							* ‡
Q1: ≤20.6	125	17	116	32	130	33	
Q2: 20.7-22.4	170	23	83	23	118	30	
Q3: 22.5-24.7	210	29	84	23	76	19	
Q4: >24.7	225	31	77	21	69	18	
Weight gain (kg) ^f^							* † ‡
Stable ^g^	80	11	31	9	65	18	
Gain, 3.0-9.9	176	25	66	19	113	31	
Gain, 10.0-19.9	234	33	91	26	96	26	
Gain, 20.0-29.9	142	20	83	24	63	17	
Gain, ≥30.0	74	11	76	22	34	9	
Waist (cm) ^d^							* † ‡
Q1: ≤ 82.3	170	22	41	14	150	43	
Q2: 82.4-90.5	211	27	65	22	77	22	
Q3: 90.6-99.8	196	25	90	30	67	19	
Q4: >99.8	195	25	102	34	57	16	
Hip (cm) ^d^							* † ‡
Q1: ≤ 100.5	206	27	48	16	103	29	
Q2: 100.6-107.5	186	24	60	20	107	31	
Q3: 107.6-116.2	183	24	85	29	87	25	
Q4: >116.2	196	25	105	35	54	15	
Waist-to-hip ratio (WHR) ^d^							* † ‡
Q1: ≤ 0.79	172	22	52	18	163	46	
Q2: 0.80-0.84	224	29	70	24	83	24	
Q3: 0.85-0.88	190	25	83	28	53	15	
Q4: >0.88	185	24	92	31	52	15	
Waist-to-height ratio (WHtR) ^d^							* †
Q1: ≤ 0.52	138	18	56	19	162	46	
Q2: 0.53-0.58	195	25	78	26	82	23	
Q3: 0.59-0.64	216	28	81	27	58	17	
Q4: >0.64	223	29	83	28	49	14	

Abbreviation: BMI, body mass index.

^a^ Chi-square test for the difference between race/ethnicity; * = *P* <0.05 between non-Hispanic Whites and Hispanics; † = *P* <0.05 between non-Hispanic Whites and African Americans; ‡ = *P* <0.05 between Hispanics and African Americans.

^b^ Percentages may not add up to 100% due to rounding.

^c^ Based on self-reported adult weight and measured height at interview (if not available, then based on measured weight at interview and/or self-reported adult height).

^d^ Based on quartiles among all postmenopausal controls.

^e^ Based on self-reported young-adult weight and measured height at interview (or self-reported adult height when measured height not available).

^f^ Self-reported adult weight (or measured weight at interview if self-reported weight not available) minus self-reported young-adult weight; excludes 64 controls who lost >3 kg of weight.

^g^ Stable weight defined as +/- 3 kg.

For women not currently using HT, associations with current BMI and adult weight gain were limited to those with ER+PR+ tumors, although after adjustment for weight gain, no association remained with current BMI (Table 3). The positive association with weight gain was not altered by adjustment for current BMI (Table 3) or young-adult BMI (data not shown), and was largely driven by the increased risk found for NHW women. Young-adult BMI was associated with reduced risk of postmenopausal BC, with similar results for BC overall (>23.7 vs. ≤21.2 kg/m^2^: OR = 0.68, 95% CI:0.54-0.86, *P*_trend_ < 0.01) and ER+PR+ BC (>23.7 vs. ≤21.2 kg/m^2^: OR = 0.73, 95% CI:0.54-0.98, *P*_trend_ = 0.04). Inverse associations, however, were found only among Hispanic and NHW women.

**Table 3:** **BMI and weight gain and breast cancer risk in postmenopausal women not currently using hormone therapy by race/ethnicity** ^**a**^ **and estrogen receptor and progesterone receptor status**

	All race/ethnicities			Hispanics			African Americans			Non-Hispanic Whites		
**All breast cancer**	**Cases (n = 801)**	**Controls (n = 1,336)**	**OR** ^**b**^ **95% CI**	**Cases (n = 377)**	**Controls (n = 709)**	**OR** ^**c**^ **95% CI**	**Cases (n = 243)**	**Controls (n = 315)**	**OR** ^**c**^ **95% CI**	**Cases (n = 181)**	**Controls (n = 312)**	**OR** ^**c**^ **95% CI**
Current BMI (kg/m^2^) ^d^												
<25.0	208	329	1.0	81	119	1.0	51	70	1.0	76	140	1.0
25.0-29.9	278	476	0.95 0.74-1.21	133	273	0.78 0.54-1.14	90	106	1.19 0.74-1.94	55	97	0.90 0.56-1.43
≥30.0	312	523	0.94 0.74-1.20	161	312	0.77 0.53-1.12	101	137	1.07 0.66-1.73	50	74	1.19 0.72-1.99
			*P*_trend_ = 0.64			*P*_trend_ = 0.24			*P*_trend_ = 0.88			*P*_trend_ = 0.58
Current BMI (kg/m^2^) –adjusted for weight gain ^d,e^												
<25.0			1.0			1.0			1.0			1.0
25.0-29.9			0.90 0.67-1.20			0.80 0.52-1.25			1.25 0.70-2.26			0.63 0.34-1.16
≥30.0			0.79 0.56-1.13			0.73 0.43-1.24			1.15 0.57-2.30			0.52 0.22-1.22
			*P*_trend_ = 0.75			*P*_trend_ = 0.26			*P*_trend_ = 0.77			*P*_trend_ = 0.11
Young-adult BMI (kg/m^2^) ^f,g^												
T1: ≤21.2	286	402	1.0	109	161	1.0	93	122	1.0	84	119	1.0
T2: 21.3-23.7	259	411	0.87 0.69-1.09	122	209	0.85 0.60-1.20	77	90	1.17 0.76-1.79	60	112	0.65 0.41-1.02
T3: >23.7	216	445	0.68 0.54-0.86	115	272	0.63 0.45-0.90	67	98	0.93 0.59-1.45	34	75	0.52 0.30-0.90
			*P*_trend_ < 0.01			*P*_trend_ = 0.01			*P*_trend_ = 0.80			*P*_trend_ = 0.01
Weight gain (kg) ^h,i^	78	140										
Stable ^j^	180	291	1.0	36	65	1.0	18	26	1.0	24	49	1.0
Gain, 3.0-9.9	217	376	1.15 0.82-1.63	82	149	1.05 0.63-1.76	50	55	1.27 0.59-2.73	48	87	1.27 0.67-2.43
Gain, 10.0-19.9	142	247	1.06 0.76-1.48	101	217	0.88 0.54-1.45	68	80	1.18 0.57-2.44	48	79	1.36 0.71-2.62
Gain, 20.0-29.9	111	154	1.03 0.72-1.48	71	125	1.04 0.61-1.78	44	72	0.91 0.43-1.93	27	50	1.19 0.57-2.48
Gain, ≥30.0			1.19 0.81-1.75	38	67	0.91 0.50-1.66	51	64	1.13 0.54-2.39	22	23	2.63 1.12-6.19
			*P*_trend_ = 0.75			*P*_trend_ = 0.75			*P*_trend_ = 0.75			*P*_trend_ = 0.10
Weight gain (kg) – adjusted for current BMI ^h,i,k^												
Stable ^j^			1.0			1.0			1.0			1.0
Gain, 3.0-9.9			1.17 0.83-1.65			1.07 0.64-1.80			1.17 0.54-2.56			1.38 0.71-2.67
Gain, 10.0-19.9			1.14 0.79-1.65			1.00 0.58-1.70			1.00 0.45-2.24			1.92 0.89-4.15
Gain, 20.0-29.9			1.17 0.77-1.78			1.22 0.66-2.25			0.76 0.32-1.85			1.94 0.75-5.03
Gain, ≥30.0			1.41 0.88-2.26			1.12 0.55-2.27			0.99 0.39-2.49			4.70 1.48-14.97
			*P*_trend_ = 0.24			*P*_trend_ = 0.67			*P*_trend_ = 0.68			*P*_trend_ = 0.02
**ER+PR+ breast cancer**	**Cases (n = 415)**	**Controls (n = 1,336)**	**OR** ^**b**^ **95% CI**	**Cases (n = 191)**	**Controls (n = 709)**	**OR** ^**c**^ **95% CI**	**Cases (n = 108)**	**Controls (n = 315)**	**OR** ^**c**^ **95% CI**	**Cases (n = 116)**	**Controls (n = 312)**	**OR** ^**c**^ **95% CI**
Current BMI (kg/m^2^) ^d^												
<25.0	98	329	1.0	34	119	1.0	19	70	1.0	45	140	1.0
25.0-29.9	141	476	1.09 0.80-1.49	60	273	0.90 0.54-1.49	44	106	1.76 0.89-3.47	37	97	1.06 0.61-1.85
≥30.0	175	523	1.30 0.95-1.78	96	312	1.23 0.75-2.01	45	137	1.46 0.74-2.89	34	74	1.39 0.76-2.55
			*P*_trend_ = 0.09			*P*_trend_ = 0.22			*P*_trend_ = 0.40			*P*_trend_ = 0.30
Current BMI (kg/m^2^) – adjusted for weight gain ^d,e^												
<25.0			1.0			1.0			1.0			1.0
25.0-29.9			1.06 0.73-1.54			1.01 0.56-1.84			1.77 0.78-4.06			0.74 0.35-1.56
≥30.0			1.01 0.64-1.60			1.17 0.58-2.35			1.29 0.48-3.48			0.64 0.23-1.76
			*P*_trend_ = 0.99			*P*_trend_ = 0.62			*P*_trend_ = 0.78			*P*_trend_ = 0.37
Young-adult BMI (kg/m^2^) ^f,g^												
T1: ≤21.2	147	402	1.0	51	161	1.0	45	122	1.0	51	119	1.0
T2: 21.3-23.7	133	411	0.87 0.65-1.15	58	209	0.87 0.55-1.37	35	90	1.23 0.69-2.20	40	112	0.66 0.38-1.15
T3: >23.7	116	445	0.73 0.54-0.98	65	272	0.80 0.51-1.25	28	98	0.73 0.39-1.36	23	75	0.52 0.27-1.00
			*P*_trend_ = 0.04			*P*_trend_ = 0.33			*P*_trend_ = 0.39			*P*_trend_ = 0.04
Weight gain (kg) ^h,l^												
Stable ^j^	44	140	1.0	18	65	1.0	9	26	1.0	17	49	1.0
Gain, 3.0-9.9	79	291	0.94 0.61-1.46	32	149	0.80 0.41-1.58	20	55	1.11 0.40-3.14	27	87	1.02 0.48-2.18
Gain, 10.0-19.9	114	376	1.07 0.71-1.62	55	217	1.00 0.53-1.88	26	80	0.96 0.36-2.57	33	79	1.48 0.70-3.15
Gain, 20.0-29.9	77	247	1.12 0.72-1.74	34	125	1.02 0.52-2.03	25	72	1.19 0.44-3.18	18	50	1.29 0.55-3.04
Gain, ≥30.0	67	154	1.53 0.96-2.45	29	67	1.43 0.70-2.94	24	64	1.27 0.47-3.42	14	23	2.56 0.95-6.88
			*P*_trend_ = 0.04			*P*_trend_ = 0.16			*P*_trend_ = 0.58			*P*_trend_ = 0.07
Weight gain (kg) – adjusted for current BMI ^h,k,l^												
Stable ^j^			1.0			1.0			1.0			1.0
Gain, 3.0-9.9			0.93 0.60-1.44			0.81 0.41-1.59			0.92 0.31-2.67			1.09 0.50-2.38
Gain, 10.0-19.9			1.04 0.66-1.65			0.96 0.49-1.89			0.67 0.22-2.04			1.90 0.77-4.73
Gain, 20.0-29.9			1.10 0.65-1.86			0.93 0.43-2.05			0.85 0.26-2.77			1.80 0.58-5.63
Gain, ≥30.0			1.53 0.85-2.73			1.29 0.55-3.01			1.02 0.29-3.58			3.82 0.99-14.71
			*P*_trend_ = 0.13			*P*_trend_ = 0.48			*P*_trend_ = 0.90			*P*_trend_ = 0.06

Abbreviations: BMI, body mass index; CI, confidence interval; ER+, estrogen receptor–positive; OR, odds ratio; PR+, progesterone receptor–positive.

^a^ All *P* values for interaction by race/ethnicity were >0.05.

^b^ Adjusted for age (years, continuous), race/ethnicity (non-Hispanic White, African American, Hispanic), place of birth (U.S.-born, foreign-born), education (some high school or less, high school or vocational/technical school graduate, some college, college graduate), first-degree family history of breast cancer (no, yes), personal history of biopsy-confirmed benign breast disease (no, yes), age at menarche (≤11, 12, 13, ≥14 years), number of full-term pregnancies (nulliparous, 1, 2, 3, ≥4), age at first full-term pregnancy (nulliparous, ≤19, 20–24, 25–29, ≥30 years), lifetime breast-feeding (nulliparous, 0, ≤6, 7–12, 13–24, ≥25 months), lifetime physical activity (quartiles, hours/week), alcohol consumption in reference year (0, 0.1-4.9, 5–9.9, 10–19.9, ≥20 g/day), and total caloric intake (quartiles, kcal/day).

^c^ Not adjusted for race/ethnicity.

^d^ Based on self-reported weight and measured height at interview (if not available, then based on measured weight at interview and/or self-reported height).

^e^ Adjusted for above variables, and weight gain.

^f^ Based on tertiles among all postmenopausal controls.

^g^ Based on self-reported young-adult weight and measured height at interview (or self-reported adult height when measured height not available).

^h^ Self-reported weight (or measured weight at interview if self-reported weight not available) minus self-reported young-adult weight.

^i^ Excludes 34 cases and 55 controls who lost >3 kg of weight.

^j^ Stable weight defined as +/- 3 kg.

^k^ Adjusted for above variables, and current BMI.

^l^ Excludes 15 ER+PR+ cases and 55 controls who lost >3 kg of weight.

Young-adult BMI was an important modifying factor (Table 4). For ER+PR+ BC, associations with high BMI (OR = 1.97, *P*_trend_ = 0.01) and high weight gain (OR = 1.71, *P*_trend_ = 0.03) were limited to women with a young-adult BMI ≤22.4 kg/m^2^. No increased risks were found among women with both high young-adult BMI and high current BMI. Associations were also influenced by time since menopause. High weight gain was associated with two-fold increased risks of BC overall (OR = 2.71, 95% CI:1.29-5.69, *P*_trend_ = 0.01) and ER+PR+ BC (OR = 2.47, 95% CI:1.03-5.94, *P*_trend_ = 0.03) only in women who had experienced menopause ≥15 years ago. Similarly, the inverse association of young-adult BMI with BC risk overall was seen only in women with ≥15 years since menopause (>23.7 vs. ≤21.2 kg/m^2^: OR = 0.59, 95% CI:0.41-0.85, *P*_trend_ < 0.01).

**Table 4:** **BMI and weight gain and breast cancer risk in postmenopausal women not currently using hormone therapy by young-adult BMI and time since menopause**

	Young-adult BMI			Young-adult BMI			
	≤22.4 kg/m^2^			>22.4 kg/m^2^			
**All breast cancer**	**Cases (n = 413)**	**Controls (n = 614)**	**OR** ^**a**^ **95% CI**	**Cases (n = 348)**	**Controls (n = 644)**	**OR** ^**a**^ **95% CI**	***P***_**interaction**_
Current BMI (kg/m^2^) ^b^							0.01
<25.0	167	243	1.0	38	77	1.0	
25.0-29.9	131	232	0.77 0.56-1.06	136	217	1.28 0.80-2.05	
≥30.0	115	138	1.22 0.86-1.74	174	350	0.92 0.58-1.45	
			*P*_trend_ = 0.42			*P*_trend_ = 0.19	
Weight gain (kg) ^c,d^							0.12
Stable ^e^	35	52	1.0	43	87	1.0	
Gain, 3.0-9.9	106	142	1.20 0.71-2.03	74	148	1.03 0.64-1.66	
Gain, 10.0-19.9	109	198	0.82 0.49-1.38	108	176	1.26 0.80-1.99	
Gain, 20.0-29.9	90	126	1.11 0.65-1.91	51	120	0.81 0.48-1.36	
Gain, ≥30.0	67	84	1.14 0.64-2.05	44	69	1.08 0.62-1.90	
			*P*_trend_ = 0.84			*P*_trend_ = 0.81	
**ER+PR+ breast cancer**	**Cases (n = 209)**	**Controls (n = 614)**	**OR** ^**a**^ **95% CI**	**Cases (n = 187)**	**Controls (n = 644)**	**OR** ^**a**^ **95% CI**	***P***_**interaction**_
Current BMI (kg/m^2^) ^b^							0.01
<25.0	77	243	1.0	19	77	1.0	
25.0-29.9	63	232	0.87 0.57-1.32	74	217	1.42 0.78-2.59	
≥30.0	69	138	1.97 1.26-3.09	94	350	1.10 0.61-1.98	
			*P*_trend_ = 0.01			*P*_trend_ = 0.69	
Weight gain (kg) ^c,f^							0.08
Stable ^e^	20	52	1.0	24	87	1.0	
Gain, 3.0-9.9	42	142	0.97 0.50-1.88	37	148	0.99 0.54-1.80	
Gain, 10.0-19.9	52	198	0.83 0.43-1.59	62	176	1.44 0.82-2.54	
Gain, 20.0-29.9	50	126	1.35 0.69-2.64	27	120	0.88 0.46-1.69	
Gain, ≥30.0	41	84	1.71 0.83-3.52	26	69	1.48 0.75-2.94	
			*P*_trend_ = 0.03			*P*_trend_ = 0.42	
	**<15 Years Since Menopause** ^**g**^			**≥15 Years Since Menopause** ^**g**^			
**All breast cancer**	**Cases (n = 335)**	**Controls (n = 552)**	**OR** ^**a**^ **95% CI**	**Cases (n = 352)**	**Controls (n = 576)**	**OR** ^**a**^ **95% CI**	***P***_**interaction**_
Current BMI (kg/m^2^) ^b^							0.09
<25.0	98	134	1.0	82	138	1.0	
25.0-29.9	115	187	0.85 0.57-1.26	126	229	0.97 0.67-1.41	
≥30.0	120	227	0.70 0.47-1.03	143	205	1.24 0.84-1.81	
			*P*_trend_ = 0.07			*P*_trend_ = 0.22	
Current BMI (kg/m^2^) –adjusted for weight gain ^b,h^							0.31
<25.0			1.0			1.0	
25.0-29.9			0.89 0.56-1.42			0.73 0.46-1.15	
≥30.0			0.85 0.47-1.51			0.68 0.39-1.18	
			*P*_trend_ = 0.58			*P*_trend_ = 0.20	
Young-adult BMI (kg/m^2^) ^i,j^							0.57
T1: ≤21.2	120	181	1.0	127	163	1.0	
T2: 21.3-23.7	112	163	1.03 0.72-1.48	112	174	0.82 0.58-1.16	
T3: >23.7	90	181	0.78 0.53-1.14	92	199	0.59 0.41-0.85	
			*P*_trend_ = 0.22			*P*_trend_ < 0.01	
Weight gain (kg) ^c,k^							0.12
Stable ^e^	33	55	1.0	32	68	1.0	
Gain, 3.0-9.9	85	116	1.13 0.65-1.96	72	133	1.29 0.76-2.20	
Gain, 10.0-19.9	101	165	0.89 0.53-1.52	92	152	1.40 0.83-2.35	
Gain, 20.0-29.9	52	98	0.82 0.46-1.48	66	105	1.44 0.83-2.50	
Gain, ≥30.0	40	73	0.69 0.37-1.30	53	55	2.09 1.15-3.81	
			*P*_trend_ = 0.08			*P*_trend_ = 0.02	
Weight gain (kg) – adjusted for current BMI ^c,k,l^							0.12
Stable ^e^			1.0			1.0	
Gain, 3.0-9.9			1.16 0.67-2.01			1.34 0.78-2.30	
Gain, 10.0-19.9			0.96 0.54-1.71			1.67 0.93-2.99	
Gain, 20.0-29.9			0.91 0.46-1.82			1.77 0.92-3.38	
Gain, ≥30.0			0.78 0.36-1.68			2.71 1.29-5.69	
			*P*_trend_ = 0.40			*P*_trend_ = 0.01	
**ER+PR+ breast cancer**	**Cases (n = 156)**	**Controls (n = 552)**	**OR** ^**a**^ **95% CI**	**Cases (n = 201)**	**Controls (n = 576)**	**OR** ^**a**^ **95% CI**	***P***_**interaction**_
Current BMI (kg/m^2^) ^b^							0.45
<25.0	42	134	1.0	40	138	1.0	
25.0-29.9	47	187	0.89 0.53-1.50	77	229	1.26 0.79-2.02	
≥30.0	66	227	1.07 0.64-1.78	84	205	1.63 1.00-2.65	
			*P*_trend_ = 0.71			*P*_trend_ = 0.05	
Current BMI (kg/m^2^) –adjusted for weight gain ^b,h^							0.43
<25.0			1.0			1.0	
25.0-29.9			0.97 0.52-1.81			1.15 0.64-2.07	
≥30.0			1.35 0.63-2.87			0.94 0.46-1.92	
			*P*_trend_ = 0.40			*P*_trend_ = 0.75	
Young-adult BMI (kg/m^2^) ^i,j^							0.76
T1: ≤21.2	56	181	1.0	67	163	1.0	
T2: 21.3-23.7	49	163	1.00 0.62-1.59	66	174	0.89 0.58-1.37	
T3: >23.7	45	181	0.89 0.54-1.46	58	199	0.70 0.45-1.10	
			*P*_trend_ = 0.64			*P*_trend_ = 0.12	
Weight gain (kg) ^c,m^							0.51
Stable ^e^	16	55	1.0	22	68	1.0	
Gain, 3.0-9.9	34	116	0.94 0.46-1.93	34	133	0.93 0.48-1.77	
Gain, 10.0-19.9	48	165	0.98 0.49-1.94	53	152	1.25 0.68-2.30	
Gain, 20.0-29.9	24	98	0.85 0.40-1.84	40	105	1.38 0.72-2.64	
Gain, ≥30.0	24	73	0.99 0.45-2.17	33	55	2.32 1.15-4.69	
			*P*_trend_ = 0.91			*P*_trend_ = 0.01	
Weight gain (kg) – adjusted for current BMI ^c,l,m^							0.48
Stable ^e^			1.0			1.0	
Gain, 3.0-9.9			0.95 0.46-1.95			0.86 0.44-1.68	
Gain, 10.0-19.9			0.94 0.44-1.97			1.17 0.59-2.33	
Gain, 20.0-29.9			0.70 0.28-1.73			1.39 0.64-3.01	
Gain, ≥30.0			0.76 0.29-1.99			2.47 1.03-5.94	
			*P*_trend_ = 0.50			*P*_trend_ = 0.03	

Abbreviations: BMI, body mass index; CI, confidence interval; ER+, estrogen receptor–positive; OR, odds ratio; PR+, progesterone receptor–positive.

^a^ Adjusted for age (years, continuous), race/ethnicity (non-Hispanic White, African American, Hispanic), place of birth (U.S.-born, foreign-born), education (some high school or less, high school or vocational/technical school graduate, some college, college graduate), family history of breast cancer in first-degree relatives (no, yes), personal history of biopsy-confirmed benign breast disease (no, yes), age at menarche (≤11, 12, 13, ≥14 years), number of full-term pregnancies (nulliparous, 1, 2, 3, ≥4), age at first full-term pregnancy (nulliparous, ≤19, 20–24, 25–29, ≥30 years), lifetime breast-feeding (nulliparous, 0, ≤6, 7–12, 13–24, ≥25 months), lifetime physical activity (quartiles, hours/week), alcohol consumption in reference year (0, 0.1-4.9, 5–9.9, 10–19.9, ≥20 g/day), and total caloric intake (quartiles, kcal/day).

^b^ Based on self-reported weight and measured height at interview (if not available, then based on measured weight at interview and/or self-reported height).

^c^ Self-reported weight (or measured weight at interview if self-reported weight not available) minus self-reported young-adult weight.

^d^ Excludes 34 cases and 55 controls who lost >3 kg of weight.

^e^ Stable weight defined as +/- 3 kg.

^f^ Excludes 15 ER+PR+ cases and 55 controls who lost >3 kg of weight.

^g^ Among women with natural or surgical menopause only.

^h^ Adjusted for above variables, and weight gain.

^i^ Based on tertiles among all postmenopausal controls.

^j^ Based on self-reported young-adult weight and measured height at interview (or self-reported adult height when measured height not available).

^k^ Excludes 28 cases and 46 controls who lost >3 kg of weight.

^l^ Adjusted for above variables, and current BMI.

^m^ Excludes 13 ER+PR+ cases and 46 controls who lost >3 kg of weight.

Waist circumference was associated with ER+PR+ BC in Hispanics (*P*_trend_ = 0.01) and AAs (*P*_trend_ = 0.05) only, with two- to three-fold increased ORs for large waist size that were independent of current BMI (Table 5). Associations were slightly stronger for ER+PR+ disease than BC overall. Large hip circumference was associated with elevated ORs in Hispanics and NHWs, with a significant trend in Hispanics (*P*_trend_ = 0.01). There was no association with WHR (data not shown). High WHtR was associated with elevated ORs in Hispanics and AAs, with a significant trend in Hispanics (*P*_trend_ = 0.01). For waist and hip circumferences and WHtR, associations did not vary by time since menopause (data not shown). Considering the joint effects of abdominal adiposity and overall adiposity (Table 6), we found that large waist circumference was associated with increased BC risk only in women with a BMI <25 kg/m^2^. A similar pattern was seen for WHtR. For ER+PR+ disease, ORs were elevated, regardless of BMI, but significant only in women with a BMI ≥25 kg/m^2^.

**Table 5:** **Abdominal adiposity and breast cancer risk in postmenopausal women not currently using hormone therapy by race/ethnicity** ^**a**^

	All race/ethnicities			Hispanics			African Americans			Non-Hispanic Whites		
**All breast cancer**	**Cases (n = 801)**	**Controls (n = 1,336)**	**OR** ^**b**^ **95% CI**	**Cases (n = 377)**	**Controls (n = 709)**	**OR** ^**c**^ **95% CI**	**Cases (n = 243)**	**Controls (n = 315)**	**OR** ^**c**^ **95% CI**	**Cases (n = 181)**	**Controls (n = 312)**	**OR** ^**c**^ **95% CI**
Waist (cm) ^d^												
T1: ≤ 85.0	198	385	1.0	96	201	1.0	30	54	1.0	72	130	1.0
T2: 85.1-96.4	214	407	0.99 0.77-1.27	113	245	0.90 0.63-1.27	59	78	1.55 0.85-2.83	42	84	0.91 0.55-1.52
T3: >96.4	293	412	1.32 1.03-1.69	146	232	1.27 0.90-1.79	102	120	1.83 1.04-3.21	45	60	1.24 0.72-2.13
			*P*_trend_ = 0.02			*P*_trend_ = 0.14			*P*_trend_ = 0.05			*P*_trend_ = 0.52
Waist (cm) – adjusted for current BMI ^d,e^												
T1: ≤ 85.0			1.0			1.0			1.0			1.0
T2: 85.1-96.4			1.09 0.82-1.44			1.08 0.73-1.60			1.64 0.84-3.20			0.80 0.44-1.47
T3: >96.4			1.59 1.15-2.19			1.79 1.14-2.81			2.17 1.05-4.49			0.90 0.42-1.91
			*P*_trend_ < 0.01			*P*_trend_ = 0.01			*P*_trend_ = 0.04			*P*_trend_ = 0.75
Hip (cm) ^d^												
T1: ≤ 102.9	194	394	1.0	105	224	1.0	39	60	1.0	50	110	1.0
T2: 103.0-112.7	230	402	1.15 0.90-1.47	119	229	1.13 0.80-1.58	54	77	1.06 0.60-1.88	57	96	1.46 0.87-2.43
T3: >112.7	281	407	1.36 1.07-1.73	131	224	1.22 0.87-1.71	98	115	1.51 0.89-2.56	52	68	1.80 1.03-3.14
			*P*_trend_ = 0.01			*P*_trend_ = 0.26			*P*_trend_ = 0.09			*P*_trend_ = 0.04
Hip (cm) – adjusted for current BMI ^d,e^												
T1: ≤ 102.9			1.0			1.0			1.0			1.0
T2: 103.0-112.7			1.27 0.97-1.66			1.34 0.92-1.94			1.04 0.54-2.00			1.58 0.90-2.79
T3: >112.7			1.66 1.20-2.30			1.64 1.05-2.58			1.68 0.82-3.42			1.88 0.86-4.10
			*P*_trend_ < 0.01			*P*_trend_ = 0.03			*P*_trend_ = 0.10			*P*_trend_ = 0.09
Waist-to-height ratio ^d^												
T1: ≤ 0.54	200	372	1.0	80	153	1.0	42	70	1.0	78	149	1.0
T2: 0.55-0.61	226	410	1.05 0.81-1.35	117	254	0.88 0.61-1.28	64	85	1.42 0.82-2.45	45	71	1.27 0.76-2.12
T3: >0.61	279	422	1.27 0.98-1.64	158	271	1.13 0.78-1.62	85	97	1.74 1.02-2.96	36	54	1.16 0.65-2.06
			*P*_trend_ = 0.06			*P*_trend_ = 0.37			*P*_trend_ = 0.05			*P*_trend_ = 0.51
Waist-to-height ratio – adjusted for current BMI ^d,e^												
T1: ≤ 0.54			1.0			1.0			1.0			1.0
T2: 0.55-0.61			1.14 0.86-1.52			1.05 0.69-1.60			1.55 0.82-2.91			1.13 0.61-2.09
T3: >0.61			1.49 1.06-2.09			1.55 0.96-2.50			2.22 1.09-4.51			0.85 0.39-1.84
			*P*_trend_ = 0.02			*P*_trend_ = 0.05			*P*_trend_ = 0.03			*P*_trend_ = 0.72
**ER+PR+ breast cancer**	**Cases (n = 415)**	**Controls (n = 1,336)**	**OR** ^**b**^ **95% CI**	**Cases (n = 191)**	**Controls (n = 709)**	**OR** ^**c**^ **95% CI**	**Cases (n = 108)**	**Controls (n = 315)**	**OR** ^**c**^ **95% CI**	**Cases (n = 116)**	**Controls (n = 312)**	**OR** ^**c**^ **95% CI**
Waist (cm) ^d^												
T1: ≤ 85.0	95	385	1.0	42	201	1.0	8	54	1.0	45	130	1.0
T2: 85.1-96.4	106	407	1.11 0.80-1.54	55	245	1.01 0.63-1.61	26	78	2.70 1.01-7.17	25	84	0.88 0.48-1.63
T3: >96.4	162	412	1.76 1.28-2.41	84	232	1.79 1.14-2.81	46	120	3.31 1.29-8.48	32	60	1.38 0.75-2.56
			*P*_trend_ < 0.01			*P*_trend_ = 0.01			*P*_trend_ = 0.02			*P*_trend_ = 0.33
Waist (cm) – adjusted for current BMI ^d,e^												
T1: ≤ 85.0			1.0			1.0			1.0			1.0
T2: 85.1-96.4			1.13 0.78-1.63			1.12 0.66-1.90			2.48 0.86-7.19			0.75 0.37-1.52
T3: >96.4			1.83 1.21-2.79			2.03 1.11-3.70			3.36 1.10-10.28			0.98 0.41-2.32
			*P*_trend_ < 0.01			*P*_trend_ = 0.01			*P*_trend_ = 0.05			*P*_trend_ = 0.93
Hip (cm) ^d^												
T1: ≤ 102.9	92	394	1.0	46	224	1.0	17	60	1.0	29	110	1.0
T2: 103.0-112.7	118	402	1.32 0.96-1.81	56	229	1.23 0.78-1.94	23	77	1.04 0.48-2.28	39	96	1.76 0.95-3.25
T3: >112.7	153	407	1.77 1.30-2.42	79	224	1.85 1.19-2.85	40	115	1.54 0.74-3.21	34	68	2.06 1.07-3.97
			*P*_trend_ < 0.01			*P*_trend_ = 0.01			*P*_trend_ = 0.20			*P*_trend_ = 0.03
Hip (cm) – adjusted for current BMI ^d,e^												
T1: ≤ 102.9			1.0			1.0			1.0			1.0
T2: 103.0-112.7			1.36 0.96-1.93			1.38 0.83-2.28			0.77 0.31-1.88			1.81 0.92-3.56
T3: >112.7			1.85 1.22-2.81			2.12 1.17-3.84			1.18 0.45-3.08			1.96 0.78-4.91
			*P*_trend_ < 0.01			*P*_trend_ = 0.01			*P*_trend_ = 0.57			*P*_trend_ = 0.12
Waist-to-height ratio ^d^												
T1: ≤ 0.54	98	372	1.0	36	153	1.0	14	70	1.0	48	149	1.0
T2: 0.55-0.61	112	410	1.15 0.83-1.59	51	254	0.88 0.53-1.46	30	85	1.94 0.86-4.35	31	71	1.44 0.79-2.65
T3: >0.61	153	422	1.61 1.16-2.23	94	271	1.63 1.02-2.62	36	97	2.19 0.99-4.82	23	54	1.14 0.59-2.23
			*P*_trend_ < 0.01			*P*_trend_ = 0.01			*P*_trend_ = 0.07			*P*_trend_ = 0.49
Waist-to-height ratio –adjusted for current BMI ^d,e^												
T1: ≤ 0.54			1.0			1.0			1.0			1.0
T2: 0.55-0.61			1.13 0.77-1.64			0.97 0.55-1.73			1.75 0.72-4.26			1.19 0.58-2.48
T3: >0.61			1.55 1.00-2.39			1.83 0.97-3.47			2.19 0.81-5.93			0.74 0.30-1.85
			*P*_trend_ = 0.04			*P*_trend_ = 0.02			*P*_trend_ = 0.14			*P*_trend_ = 0.62

Abbreviations: BMI, body mass index; CI, confidence interval; ER+, estrogen receptor–positive; OR, odds ratio; PR+, progesterone receptor–positive.

^a^ All *P* values for interaction by race/ethnicity were >0.05.

^b^ Adjusted for age (years, continuous), race/ethnicity (non-Hispanic White, African American, Hispanic), place of birth (U.S.-born, foreign-born), education (some high school or less, high school or vocational/technical school graduate, some college, college graduate), first-degree family history of breast cancer (no, yes), personal history of biopsy-confirmed benign breast disease (no, yes), age at menarche (≤11, 12, 13, ≥14 years), number of full-term pregnancies (nulliparous, 1, 2, 3, ≥4), age at first full-term pregnancy (nulliparous, ≤19, 20–24, 25–29, ≥30 years), lifetime breast-feeding (nulliparous, 0, ≤6, 7–12, 13–24, ≥25 months), lifetime physical activity (quartiles, hours/week), alcohol consumption in reference year (0, 0.1-4.9, 5–9.9, 10–19.9, ≥20 g/day), and total caloric intake (quartiles, kcal/day).

^c^ Adjusted for above variables, except race/ethnicity.

^d^ Based on tertiles among all postmenopausal controls.

^e^ Adjusted for above variables, and current BMI.

**Table 6:** **Abdominal adiposity and breast cancer risk in postmenopausal women not currently using hormone therapy by current BMI**

	Current BMI < 25.0kg/m^2^			Current BMI ≥ 25.0kg/m^2^			
**All breast cancer**	**Cases (n=396)**	**Controls (n=648)**	**OR** ^**a**^ **95% CI**	**Cases (n=402)**	**Controls (n=680)**	**OR** ^**a**^ **95% CI**	***P***_**interaction**_
Waist (cm) ^b^							
M1: ≤ 90.5	151	272	1.00	140	321	0.87 0.65-1.18	0.25
M2: > 90.5	27	26	1.90 1.05-3.44	387	585	1.21 0.93-1.57	
Waist-to-height ratio ^b^							
M1: ≤ 0.58	156	276	1.00	152	310	0.93 0.69-1.24	0.93
M2: > 0.58	22	22	1.87 0.98-3.56	375	596	1.17 0.90-1.52	
**ER+PR+ breast cancer**	**Cases (n=194)**	**Controls (n=648)**	**OR** ^**a**^ **95% CI**	**Cases (n=220)**	**Controls (n=680)**	**OR** ^**a**^ **95% CI**	***P***_**interaction**_
Waist (cm) ^b^							
M1: ≤ 90.5	73	272	1.00	67	321	0.92 0.62-1.36	0.16
M2: > 90.5	10	26	1.53 0.69-3.39	213	585	1.55 1.11-2.17	
Waist-to-height ratio ^b^							
M1: ≤ 0.58	75	276	1.00	75	310	1.02 0.70-1.50	0.47
M2: > 0.58	8	22	1.53 0.63-3.68	205	596	1.52 1.08-2.12	

Abbreviations: BMI, body mass index; CI, confidence interval; ER+, estrogen receptor–positive; OR, odds ratio; PR+, progesterone receptor–positive.

^a^ OR and 95% CI adjusted for age (years, continuous), race/ethnicity (non-Hispanic White, African American, Hispanic), place of birth (US-born, foreign-born), education (some high school or less, high school or vocational/technical school graduate, some college, college graduate), family history of breast cancer in first-degree relatives (no, yes), personal history of biopsy-confirmed benign breast disease (no, yes), age at menarche (≤11, 12, 13, ≥14 years), number of full-term pregnancies (nulliparous, 1, 2, 3, ≥4), age at first full-term pregnancy (nulliparous, ≤19, 20–24, 25–29, ≥30 years), lifetime breast-feeding (nulliparous, 0, ≤6, 7–12, 13–24, ≥25 months), lifetime physical activity (quartiles, hours/week), alcohol consumption in reference year (0, 0.1-4.9, 5–9.9, 10–19.9, ≥20 g/day), and total caloric intake (quartiles, kcal/day).

^b^ Based on median among all postmenopausal controls.

For ER-PR- BC, there were no associations with current BMI and weight gain, whereas a strong inverse association was found with young-adult BMI (>23.7 vs. ≤21.2 kg/m^2^: OR = 0.61, 95% CI:0.38-0.97, *P*_trend_ = 0.04) (Table 7). Modest positive associations with waist and hip circumferences were strengthened after adjustment for current BMI (*P*_trend_ = 0.07 and 0.01, respectively). Sample sizes of ER-PR- cases were too small for further stratification by race/ethnicity (9 NHWs, 48 AAs, 79 Hispanics).

**Table 7:** **Body size and risk of ER-PR- breast cancer in postmenopausal women not currently using hormone therapy**

	Cases (n=135)	Controls (n=1,336)	OR^a^, 95% CI	OR^b^95% CI	OR^c^95% CI
Current BMI(kg/m^2^) ^d^					
<25.0	34	329	1.00		1.00
25.0-29.9	46	476	0.75 0.46-1.22		0.62 0.36-1.08
≥30.0	54	523	0.72 0.45-1.16		0.58 0.30-1.14
			*P*_trend_ = 0.21		*P*_trend_ = 0.13
Young-adult BMI (kg/m^2^) ^e,f^					
T1: ≤21.2	46	402	1.00		
T2: 21.3-23.7	43	411	0.82 0.52-1.29		
T3: >23.7	37	445	0.61 0.38-0.97		
			*P*_trend_ = 0.04		
Weight gain (kg) ^g^					
Stable ^h^	10	140	1.00	1.00	
Gain, 3.0-9.9	31	291	1.38 0.65-2.93	1.47 0.69-3.16	
Gain, 10.0-19.9	42	376	1.31 0.63-2.72	1.67 0.76-3.65	
Gain, ≥20.0	39	401	1.05 0.50-2.19	1.45 0.60-3.48	
			*P*_trend_ = 0.63	*P*_trend_ = 0.48	
Waist (cm) ^e^					
T1: ≤ 85.0	28	385	1.00	1.00	
T2: 85.1-96.4	40	407	1.13 0.67-1.89	1.43 0.80-5.54	
T3: >96.4	48	412	1.24 0.75-2.06	1.87 0.96-3.64	
			*P*_trend_ = 0.41	*P*_trend_ = 0.07	
Hip (cm) ^e^					
T1: ≤ 102.9	27	394	1.00	1.00	
T2: 103.0-112.7	41	402	1.40 0.84-2.34	1.85 1.05-3.28	
T3: >112.7	48	407	1.43 0.86-2.37	2.35 1.20-4.59	
			*P*_trend_ = 0.19	*P*_trend_ = 0.01	
Waist-to-hip ratio					
T1: ≤ 0.81	33	434	1.00	1.00	
T2: 0.82-0.86	34	355	1.14 0.68-1.90	1.21 0.72-2.04	
T3: >0.86	49	413	1.35 0.83-2.18	1.46 0.88-2.39	
			*P*_trend_ = 0.22	*P*_trend_ = 0.14	
Waist-to-height ratio					
T1: ≤ 0.54	30	372	1.00	1.00	
T2: 0.55-0.61	40	410	0.98 0.59-1.63	1.19 0.66-2.17	
T3: >0.61	46	422	1.05 0.63-1.73	1.44 0.72-2.87	
			*P*_trend_ = 0.84	*P*_trend_ = 0.30	

Abbreviations: BMI, body mass index; CI, confidence interval; ER-, estrogen receptor–negative; OR, odds ratio; PR-, progesterone receptor–negative.

^a^ OR and 95% CI adjusted for age (years, continuous), race/ethnicity (non-Hispanic White, African American, Hispanic), place of birth (US-born, foreign-born), age at menarche (≤11, 12, 13, ≥14 years), and lifetime breast-feeding (nulliparous, 0, ≤6, 7–12, 13–24, ≥25 months).

^b^ Adjusted for above variables, and current BMI.

^c^ Adjusted for above variables, and weight gain.

^d^ Based on self-reported weight and measured height at interview (if not available, then based on measured weight at interview and/or self-reported height).

^e^ Based on tertiles among all postmenopausal controls.

^f^ Based on self-reported young-adult weight and measured height at interview (or self-reported adult height when measured height not available).

^g^ Self-reported weight (or measured weight at interview if self-reported weight not available) minus self-reported young-adult weight; excludes 5 cases and 55 controls who lost >3 kg of weight

^h^ Stable weight defined as +/- 3 kg.

Among women currently using HT (289 cases, 498 controls), there was no evidence of significant associations between any of the body size measures examined and BC risk overall or ER+PR+ disease (data not shown).

## Discussion

In postmenopausal women not currently using HT, weight gain was positively associated with risk of ER+PR+ BC and was a stronger predictor of risk than current BMI. The highest elevations in risks were found in subgroups of women with a low young-adult BMI or ≥15 years since menopause. Young-adult obesity was associated with reduced BC risk. High waist circumference and WHtR were associated with increased BC risk, independent of current BMI. Associations with weight gain and young-adult BMI were stronger for NHWs than Hispanics and AAs, whereas associations with waist and WHtR were present only in Hispanic and AA women.

Consistent with other reports (White et al. 2012; Huang et al. 1997; Ahn et al. 2007; Feigelson et al. 2004), we found that weight gain was an important risk factor for postmenopausal BC, independent of current BMI. For current BMI no association remained after adjustment for weight gain. In agreement with other studies (Vrieling et al. 2010), we found that the relation with weight gain was limited to ER+PR+ BC. Risk was increased two-fold for currently obese women (BMI ≥30 kg/m^2^) who had a young-adult BMI <22.4 kg/m^2^, which is in agreement with other studies (Ahn et al. 2007; Canchola et al. 2012). We found modest effect modification by young adult BMI for weight gain, though some other studies did not (Barnes-Josiah et al. 1995; Feigelson et al. 2004; van den Brandt et al. 1997; Lahmann et al. 2005). In contrast, BC risk was not increased in women who were obese throughout their adult life, consistent with other (Ahn et al. 2007; Canchola et al. 2012), but not all reports (Barnes-Josiah et al. 1995). In agreement with other reports (Chu et al. 1991; Magnusson et al. 1998; Macinnis et al. 2004), we found time since menopause to be another important modifying factor, with two-fold increased risks of ER+PR+ BC with weight gain only among women with ≥15 years since menopause. Weight gain during adulthood largely reflects an increase in body fat which serves as an important source of estrogen production in postmenopausal women (Siiteri 1987). The role of an estrogen-related pathway is further supported by the observation that the associations with BMI and weight gain are limited to ER+PR+ tumors (Suzuki et al. 2009; Vrieling et al. 2010).

Prior findings in AA and Hispanic women for BMI are inconsistent. In our study, OR estimates were increased only for ER+PR+ disease and were of similar magnitude in the three racial/ethnic groups. Two studies in AAs reported elevated risks of ER+PR+ disease (Palmer et al. 2007; Berstad et al. 2010), and, similar to our study, there was no evidence of an association with BMI for BC overall. Other studies did not consider hormone receptor status (White et al. 2012; Schatzkin et al. 1987; Adams-Campbell et al. 1996; Hall et al. 2000; Zhu et al. 2005), and not all found a positive association with BMI (White et al. 2012; Schatzkin et al. 1987; Zhu et al. 2005). In black women from Nigeria (Ogundiran et al. 2010; Okobia et al. 2006; Adebamowo et al. 2003) and Barbados (Nemesure et al. 2009), no associations with BMI were found. In Hispanic women, BMI was not associated with BC overall (Wenten et al. 2002; Slattery et al. 2007; White et al. 2012) and ER+ disease (Slattery et al. 2007), even among women not using HT (Slattery et al. 2007; White et al. 2012).

We found that high weight gain was associated with a two-fold increased risk of ER+PR+ BC in NHW women. In AAs and Hispanics, the associations were much weaker, likely due to the higher prevalence of young-adult obesity in these groups. Of three studies in AAs that reported on weight gain and BC risk (White et al. 2012; Zhu et al. 2005; Palmer et al. 2007), only one found a significant association with BC risk overall (White et al. 2012). In Hispanic women from New Mexico, large weight gain was associated with a two-fold increased risk of ER+PR+ disease and, for BC overall, a significant trend with weight gain was limited to women with a BMI <22 kg/m^2^ at age 18 years (Wenten et al. 2002). Two other studies in Hispanics found no association with weight gain (Slattery et al. 2007; White et al. 2012). In order to address these inconsistent findings with BMI and weight gain for AA and Hispanic women, future studies should examine the modifying effect of young-adult obesity with larger sample sizes. This is particularly important since AA and Hispanic women have a higher prevalence of young-adult obesity than NHWs, as found in our study as well as others (Flegal et al. 2010).

Our finding of a strong inverse association of postmenopausal BC risk with high young-adult BMI, which was independent of weight change or current BMI, is consistent with other reports (White et al. 2012; Palmer et al. 2007; Berstad et al. 2010; Barnes-Josiah et al. 1995; Huang et al. 1997; Morimoto et al. 2002; Ahn et al. 2007; Chu et al. 1991; Brinton & Swanson 1992; Magnusson et al. 1998), although in some studies there was no association with young-adult BMI (Canchola et al. 2012; Feigelson et al. 2004; Lahmann et al. 2005). In agreement with a large meta-analysis (Suzuki et al. 2009), we found that the inverse association with young-adult BMI did not differ by tumor hormone receptor status. We further found an inverse association, regardless of HT use, as reported by others (Morimoto et al. 2002; Ahn et al. 2007). These findings do not support an estrogen-related mechanism underlying the association with young-adult BMI.

Abdominal adiposity has been proposed to be more important in estrogen production than adiposity at other body sites (Pinheiro et al. 2009). Studies in primarily NHW women, however, have produced inconsistent results (World Cancer Research Fund / American Institute for Cancer Research 2007). Not all studies considered HT use or ER/PR status, or adjusted for overall adiposity (Canchola et al. 2012; Potter et al. 1995; Huang et al. 2000). Our findings confirm previous reports of positive associations with waist circumference and WHtR only in women not currently using HT (Morimoto et al. 2002; Huang et al. 1999; Friedenreich et al. 2002) and stronger associations for ER+PR+ disease (Canchola et al. 2012; Potter et al. 1995; Huang et al. 2000). We found no association with WHR, whereas elevated WHtR was associated with increased risk, in agreement with another study (Canchola et al. 2012). Both waist circumference and WHtR may be better measures of abdominal adiposity than WHR (Molarius & Seidell 1998; Rankinen et al. 1999). In some studies, the association with abdominal adiposity was attenuated after adjustment for BMI (Morimoto et al. 2002; Lahmann et al. 2004; Tehard & Clavel-Chapelon 2006), whereas in our study associations became stronger after BMI-adjustment. Among women with BMI <25 kg/m^2^, large waist circumference and high WHtR were associated with two-fold increased risks of BC overall. Contrary to another study that reported an association between abdominal adiposity and ER+PR+ tumors only in normal-weight women (Canchola et al. 2012), we found elevated ORs for ER+PR+ tumors, regardless of BMI, with statistically significant estimates in overweight/obese women.

Unlike NHW women, for whom we found no associations with waist circumference and WHtR, AAs and Hispanics had two- to three-fold increased risk of ER+PR+ BC. We found no association with WHR in any racial/ethnic group. This latter finding is consistent with two studies in AA women (Hall et al. 2000; Palmer et al. 2007), but contrasts with reports from Nigeria (Ogundiran et al. 2010) and Barbados (Nemesure et al. 2009), where large waist circumference and high WHR increased BC risk. Similarly, the two-fold increased risk of ER+PR+ disease associated with large hip circumference that we observed for NHW and Hispanic women was not seen in AAs, whereas previous studies from Nigeria (Ogundiran et al. 2012) and Barbados (Nemesure et al. 2009) reported inverse associations with hip circumference. In the Nigerian study, associations with abdominal adiposity were stronger or limited to women with a BMI <25 kg/m^2^ (Ogundiran et al. 2012), consistent with our findings. In the only study that examined abdominal adiposity in postmenopausal Hispanic women, hip circumference and WHR were not associated with BC risk in not recent HT users (Slattery et al. 2007), which differs from our finding. Given these conflicting reports, it remains to be determined whether there are true racial/ethnic differences in the effects of abdominal adiposity on BC risk. Abdominal fat comprises different fat stores, and AAs and NHWs differ in abdominal depot-specific body fat (e.g., visceral vs. subcutaneous adipose tissue) (Katzmarzyk et al. 2010). Whether different fat stores affect BC risk differentially has not been examined. Our results suggest that studies should assess multiple measures of adiposity in racially/ethnically diverse populations.

Our analyses of body size and ER-PR- BC risk were limited by small numbers. Consistent with most other studies (Suzuki et al. 2009; Yang et al. 2011), we found no associations with current BMI and weight gain, although there are some reports of inverse (Berstad et al. 2010; Setiawan et al. 2009) or positive associations with BMI (Ritte et al. 2012) and positive associations with weight gain (Canchola et al. 2012). Unlike other studies (Canchola et al. 2012; Potter et al. 1995), we found a strong inverse association with young-adult BMI and ER-PR- disease. Adjustment for BMI strengthened the positive association between waist (*P*_trend_ = 0.07) and hip (*P*_trend_ = 0.01) circumferences and risk of ER-PR- BC, but, consistent with other studies (Canchola et al. 2012; Potter et al. 1995; Huang et al. 2000), we found no significant association with WHR or WHtR. The association between BMI, weight gain, and abdominal adiposity warrants further examination in studies with larger numbers of ER-PR- cases. This is particularly important since few risk factors have been identified for hormone receptor negative BC (Althuis et al. 2004; Ma et al. 2006), which disproportionately affects AA and Hispanic women (Ray & Polite 2010).

Our findings should be considered in light of some limitations. Due to the concern that weight may be impacted by BC diagnosis and treatment, we relied on self-reported weight during the reference year. Although we cannot exclude the possibility of inaccurately recalled weight, among subjects for whom measured and self-reported weight was available, the correlation between the two measures was high (*r* = 0.85 for cases, *r* = 0.92 for controls). For waist and hip circumference we had to rely on measurements taken after diagnosis which may have also resulted in misclassification. Finally, some subgroup analyses were limited by small sample sizes, and analyses of potential modifying factors (young-adult BMI, time since menopause) could not be further stratified by race/ethnicity. Larger studies or analyses of pooled data will be necessary to further explore the role of these modifying factors in Hispanics and AAs.

Our study also has several important strengths, including a population-based design, high participation rates among cases and controls in each racial/ethnic group, assessment of both overall and abdominal adiposity, detailed data on established BC risk factors, and availability of data on tumor hormone receptor status for most cases. The racial/ethnic diversity of the study population allowed us to assess associations with body size in Hispanic and AA women, thus contributing to the relatively sparse data in these two racial/ethnic populations that experience a greater burden of obesity than NHWs.

It has been estimated that as many as one third of new postmenopausal BC cases may be attributable to adult weight gain (Huang et al. 1997). Given that a number of BC risk factors relate to events well before menopause (e.g., age at menarche, age at first birth) or characteristics that cannot easily be modified (e.g., BC family history), observed associations with body size suggest possible approaches to lowering BC risk in older women through weight maintenance, avoidance of further weight gain and abdominal adiposity, or weight loss. However, promoting overweight at a young age, as a means of reducing BC risk after menopause, should not be encouraged, given the many adverse health effects associated with obesity, including other cancers (Calle & Kaaks 2004; Chen et al. 2011).

## Abbreviations

AA: African American
BC: Breast cancer
BMI: Body mass index
CI: Confidence interval
ER: Estrogen receptor
HT: Hormone therapy
OR: Odds ratio
NHW: non-Hispanic white
PR: Progesterone receptor
WHR: Waist-to-hip ratio
WHtR: Waist-to-height ratio.
